# Gastric cancer risk is reduced by a predominance of antioxidant factors in the oxidative balance: a hospital-based case-control study in Korea

**DOI:** 10.4178/epih.e2022089

**Published:** 2022-10-17

**Authors:** Jimi Kim, Jeonghee Lee, Il Ju Choi, Young-Il Kim, Jeongseon Kim

**Affiliations:** 1 Department of Cancer Biomedical Science, Graduate School of Cancer Science and Policy, National Cancer Center, Goyang, Korea; 2 Center for Gastric Cancer, National Cancer Center Hospital, National Cancer Center, Goyang, Korea

**Keywords:** Gastric cancer, *Helicobacter pylori*, Oxidative balance score, Antioxidants, Pro-oxidants

## Abstract

**OBJECTIVES:**

Gastric carcinogenesis is linked to oxidative stress from both exogenous and endogenous exposures. This study aimed to determine the association between the risk of gastric cancer and the oxidative balance score (OBS), which comprises antioxidant and pro-oxidant factors, including diet and lifestyle.

**METHODS:**

For this hospital-based case-control study, 808 controls and 404 patients with gastric cancer who had clinical records indicating *Helicobacter pylori* infection and the histological subtype of cancer were recruited. The OBS was determined based on diet and lifestyle factors obtained from a 106-item semiquantitative food frequency questionnaire and a constructed questionnaire. Logistic regression analysis was used to estimate odds ratios (ORs) and 95% confidence intervals (CIs).

**RESULTS:**

A higher OBS was associated with a reduced gastric cancer risk (OR, 0.49; 95% CI _T3 vs. T1,_ 0.33 to 0.71; p for trend <0.001). In a subgroup analysis, antioxidant factors showed inverse associations with gastric cancer risk (OR, 0.53; 95% CI _T3 vs. T1,_ 0.35 to 0.79; p for trend=0.003). A stronger association with antioxidant factors was observed in patients with intestinal gastric cancer (OR, 0.34; 95% CI _T3 vs. T1,_ 0.19 to 0.62; p for trend<0.001) and those with *H. pylori* infection (OR, 0.57; 95% CI _T3 vs. T1,_ 0.37 to 0.88; p for trend=0.014).

**CONCLUSIONS:**

A predominance of antioxidant factors compared to pro-oxidant factors from diet and lifestyle reduced the risk of gastric cancer. The combined effect of oxidative stress, which involves an altered balance between antioxidants and pro-oxidants, is important for modulating the risk of gastric cancer.

## GRAPHICAL ABSTRACT

[Fig f2-epih-44-e2022089]

## INTRODUCTION

In 2020, gastric cancer (GC) ranked fifth in incidence (a total of 1,089,103 new GC cases) and fourth in mortality (a total of 768,793 deaths) worldwide [1]. In geographically diverse countries, GC is a commonly diagnosed cancer, and high incidence rates of GC among both men and women are observed in East Asia, including the Korea [2]. The most well-known risk factor for gastric carcinogenesis is *Helicobacter pylori* (*H. pylori*) infection, and environmental factors may exert different effects on various GC subsites or histological types [3]. Although evidence regarding dietary risk factors is limited, etiological nutritional factors remain important [4].

Regarding GC pathogenesis, reactive oxygen species (ROS) produced by cells under oxidative stress contribute to carcinogenesis [5]. Increased levels of ROS promote the initiation and progression of GC by inducing inflammation, but the underlying mechanism remains unclear [6,7]. ROS generation occurs in response to stimuli from a variety of environmental factors such as diet, nutrients, smoking, alcohol consumption, and physical activity [8,9]. ROS are generated by dietary and lifestyle factors, since increasing the postprandial metabolic rate and cigarette smoking generate a series of oxidants [8]. According to a recent study, muscle activity during exercise regulates the generation and level of ROS [10]. In physiological processes, ROS produced by unhealthy eating habits or an unbalanced lifestyle exert direct and indirect effects on growth factor signaling, the hypoxia response, inflammation, and the immune response [9]. A relative excess of ROS leads to an imbalance between the generation and accumulation of ROS and the levels of endogenous and exogenous antioxidants. An imbalance between pro-oxidants and antioxidants results in lipid peroxidation, protein oxidation, and DNA damage, stimulating tumorigenesis or supporting the proliferation of cancer cells [5,11]. Epidemiological studies have shown that among the individual components associated with GC development, certain modifiable factors that may affect GC risk play a role in oxidative stress as risk factors for the production of ROS or as preventive factors that promote an increased antioxidant capacity [3,12].

According to the evidence-based literature and rationales for individual exposures that affect oxidative stress, a combination of exposures, which include dietary and nondietary lifestyle factors, was developed to generate the oxidative balance score (OBS), which comprises pro-oxidant and antioxidant factors [13]. Given the correlations among cancer initiation, oxidative stress, and environmental exposures, the OBS, which consists of diet and lifestyle factors that mediate the regulation of ROS levels, is thought to account for the associated cancer risk [14,15]. A few epidemiological studies have investigated the associations between the OBS and all-cause and cancer-related mortality rates for patients with different types of cancer (breast, prostate, lung, and colorectal cancer) [16]. Although the associations between GC risk and individual components have been investigated on the basis of inflammatory mechanisms, there has not yet been an epidemiological study with an approach that comprehensively considers the oxidative stress exposures leading to oxidative imbalance.

In this study, we constructed the OBS with a variety of pro-oxidant and antioxidant factors comprising fat, vitamins, minerals, carotenoids, flavonoids, fiber, specific foods, body mass index (BMI), alcohol consumption, smoking, and physical activity to determine the association between the oxidative balance and GC risk. We examined individuals’ pro-oxidant dietary and lifestyle factors, as well as their antioxidant dietary and lifestyle factors. We hypothesized that combined exposure to factors that are related to oxidative stress would affect the regulation of ROS levels, mediating GC risk.

## MATERIALS AND METHODS

### Study population

**Figure 1.**
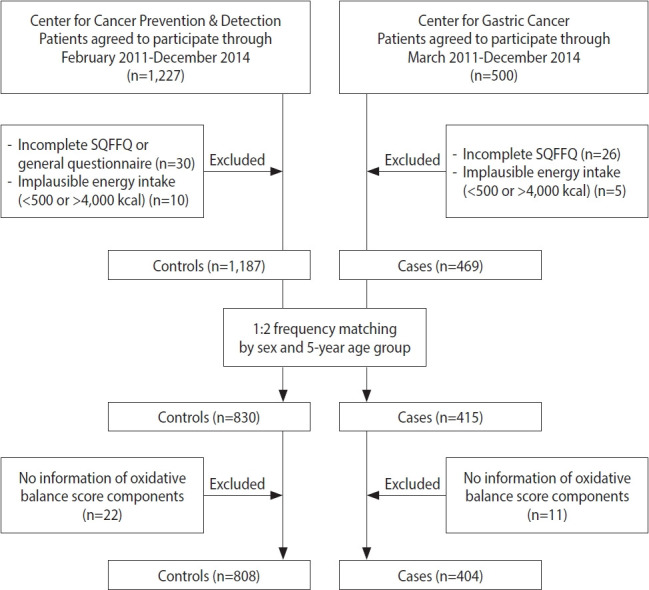
Flow chart of the study subjects. SQFFQ, semiquantitative food frequency questionnaire.

For the current hospital-based case-control study, the participants were recruited at the Center for Gastric Cancer and the Center for Cancer Prevention & Detection of the National Cancer Center in Korea between February 2011 and December 2014. Five hundred patients who were newly diagnosed with early-stage GC that was histologically confirmed as invasive carcinoma were enrolled. Among them, 31 patients were excluded due to an incomplete semiquantitative food frequency questionnaire (SQFFQ) and implausible energy intake (< 500 or > 4,000 kcal/day). During the same time period, subjects who had visited the same hospital for a health checkup program were recruited for the control group. Of 1,227 subjects, 40 controls with an incomplete SQFFQ and implausible energy intake (< 500 or > 4,000 kcal/day) were excluded, and the others were frequency-matched to cases (1:2 ratio of cases vs. controls) by age (5-year groups) and sex. Within the case and control groups, subjects missing the data required to calculate the OBS were excluded (11 cases and 22 controls). Consequently, 1,212 individuals, comprising 404 cases and 808 controls, participated in this study (Figure 1).

### Data collection and *Helicobacter pylori* infection

Socio-demographic characteristics were collected from a self-administered questionnaire. BMI was calculated from the subjects’ current height and weight, which were measured with standardized equipment. *H. pylori* infection was histologically or serologically detected with a rapid urease test (Pronto Dry, Medical Instruments Corp., Solothurn, Switzerland), resulting in either a positive or negative infection status.

### Assessment of oxidative balance score

A 106-item SQFFQ was used by a well-trained interviewer to collect dietary information. The validity and reproducibility of the SQFFQ developed for assessing the habitual diet and nutrient intake in the Korean population were evaluated previously [17]. The SQFFQ was constructed based on the frequency of consumption (daily, weekly, and monthly patterns) and the portion sizes (small, medium, and large) of food items. Daily energy and nutrient intake were analyzed using CAN-PRO 4.0 (Computer Aided Nutritional analysis program, Korean Nutrition Society, Seoul, Korea). Food items were merged according to the database of carotenoid contents developed in this study using the United States Department of Agriculture (USDA) carotenoid database, and the Korean functional food composition table contained 2,903 food items [18]. The carotenoid subclasses included α-carotene, β-carotene, lycopene, β-cryptoxanthin, and lutein/zeaxanthin. The total flavonoid intake was estimated using a flavonoid database constructed from the USDA flavonoid database, the Korean functional food composition table, and the Japanese functional food factor database [19]. The subclass of flavonoids included flavonols, flavones, flavanones, flavan-3-ols, anthocyanidins, and isoflavones. The values of flavonoids and their subclasses were reported as aglycone forms and total flavonoid intake was calculated as the sum of intake of the flavonoid subclasses described above.

The OBS, which comprises 26 components (22 dietary factors and 4 lifestyle factors), was developed based on a literature review of the association between the OBS and chronic diseases [16]. The components of the OBS were classified as pro-oxidant or antioxidant factors (Supplementary Material 1). All food and nutrient intakes were adjusted for energy intake using a residual regression method [20]. The pro-oxidant components included the intake of total fat, ω-6 fatty acids, saturated fatty acids, iron, red and processed meats, alcohol consumption and smoking statuses (current, former, and none), and BMI (normal: 18.5-22.9, overweight: 23.0- 24.9, and obese: ≥ 25.0 kg/m^2^). The antioxidant components included the intake of vitamins A, D, E, and C, folate, carotenoid subclasses (α-carotene, β-carotene, lycopene, β-cryptoxanthin, and lutein/zeaxanthin), ω-3 fatty acids, selenium, zinc, calcium, total flavonoids, fiber, cruciferous vegetables (broccoli, cabbage, kale, bok choy, radish, mustard, horseradish, and shepherd’s purse), and regular physical activity. A standardized International Physical Activity Questionnaire short form scoring protocol (https://www.physiopedia.com/images/c/c7/Quidelines_for_interpreting_the_IPAQ.pdf) was used to measure the physical activity using metabolic equivalents for task (METs), which estimated the amount of energy expenditure in a typical week or day based on intensity, frequency, and duration [21]. The MET levels for different types of activities were light-intensity activity (3.3 METs), moderate-intensity aerobic physical activity (4.0 METs), and vigorous-intensity aerobic physical activity (8.0 METs). The method of equal weight was used to estimate the individual components of the OBS, assuming that all components were equal (i.e., with the same importance) [22,23]. The continuous dietary intake variables were divided into tertiles based on the daily consumption of each. In terms of pro-oxidant components, participants with a high intake of pro-oxidant factors were classified into the highest tertile group and were assigned scores of 0 points, while subjects in the lowest tertile group were allocated scores of 2 points. Regarding the categorical pro-oxidant lifestyle factor variables, alcohol consumption and smoking status (current, former, and none) and BMI (obese: ≥ 25.0, overweight: 23.0-24.9, and normal: 18.5-22.9 kg/m^2^) were assigned scores of 2 points, 1 point, and 0 points, respectively. Participants with the highest intake of dietary antioxidant components were allocated 2 points, whereas those with the lowest intake were assigned 0 points. Regarding physical activity, participants who exercised regularly were categorized into the highest tertile group of METs/wk and assigned 2 points, while those who rarely exercised were assigned to the lowest tertile group and assigned 0 points. The total oxidative balance score (TOBS) was defined as the sum of the scores for each component.

### Statistical analysis

General demographic characteristics and TOBS were compared between the patients and controls using the t-test for continuous variables and the chi-square test for categorical variables. A logistic regression analysis was performed, adjusting for the potential confounding factors, such as education level, income, sodium intake, a first-degree family history of GC, and *H. pylori* infection, as identified by backward selection using risk factors based on previous evidence and the present study to estimate odds ratios (ORs) and their corresponding 95% confidence intervals (CIs) [3,24,25]. Multinomial logistic regression was used for the histological subtype of GC. Statistical analysis was performed using the SAS version 9.4 (SAS Institute Inc., Cary, NC, USA). Two-sided p-values less than 0.05 were regarded as indicating statistical significance.

### Ethics statement

This study was conducted according to the guidelines established in the Declaration of Helsinki, and all procedures involving participants in this research study were approved by the Institutional Review Board of the National Cancer Center Korea. All participants provided written informed consent, and the Institutional Review Board of the National Cancer Center Korea approved the study (IRB No. NCC2021-0181).

## RESULTS

### General characteristics of study subjects

Table 1 shows the differences in demographics, lifestyle factors, and relevant aspects of the OBS between patients with GC and controls. They did not differ in age or sex since the cases and controls in this study were matched. Moreover, no significant BMI differences were found between them. Statistically significant differences in education level, occupation, and income were observed (p< 0.001). Patients with GC were more likely to have a positive *H. pylori* infection and a first-degree family history of GC than the controls (p< 0.001). In terms of lifestyle factors, statistically significant differences in smoking status and regular physical activity (p< 0.001) but not in the alcohol consumption status were observed. Patients with GC were more likely to smoke and less likely to engage in regular physical activity (p< 0.001). The GC group had significantly higher intake of daily total energy than the control group (p< 0.001). When comparing the relevant oxidative balance scores between the groups, the TOBS was lower among patients with GC than among the controls (p < 0.001). Among the subclasses of the TOBS, patients with GC had significantly increased scores for pro-oxidant dietary factors (p= 0.043). However, the total antioxidant scores, which included diet and lifestyle factors, were significantly lower for patients with GC than for the controls (p< 0.001). The histological subtypes of GC were intestinal (38.6%), diffuse (38.9%), mixed (14.4%), and intermediate (0.7%).

### Comparison of individual components of the oxidative balance score

Table 2 presents comparisons of each component of the oxidative balance score between the groups. Among the pro-oxidant dietary factors, lower intake of iron was observed among patients with GC than among the controls (p= 0.003). Regarding the pro-oxidant lifestyle factors, smoking was significantly more common among patients with GC than among controls (p< 0.001). In terms of the antioxidant dietary factors, patients with GC had significantly lower intake of vitamins (A [p= 0.027], D [p< 0.001], E [p< 0.001], folate [p = 0.018], and C [p < 0.001]), subclasses of carotenoids (β-carotene [p= 0.021], lycopene [p< 0.001], and β-cryptoxanthin [p=0.008]), polyunsaturated fatty acids (ω-3 fatty acids [p=0.031]), calcium (p= 0.004), flavonoids (p= 0.001), fiber (p= 0.006), and cruciferous vegetables (p= 0.009) than the controls. With respect to antioxidant lifestyle factors, patients with GC less frequently performed regular exercise than the controls (p< 0.001).

### Association between oxidative balance score and gastric cancer risk

Table 3 indicates the association between GC risk and the OBS with its subclasses. GC risk was significantly lower in participants who had a higher TOBS in model II after adjusting for education, income, sodium intake, and first-degree family history of GC (OR, 0.41; 95% CI _T3 vs. T1_, 0.28 to 0.58; p for trend < 0.001). With additional adjustment for *H. pylori* infection in model III, inverse associations were observed between the TOBS and GC risk (OR, 0.49; 95% CI _T3 vs. T1_, 0.33 to 0.71; p for trend < 0.001). In the subclasses, the score for pro-oxidant lifestyle factors showed an association with GC risk in model II (OR, 1.49; 95% CI _T3 vs. T1_, 1.05 to 2.11; p for trend=0.028). However, a high total antioxidant score was significantly associated with reduced GC risk compared with a low total antioxidant score in both model II (OR, 0.46; 95% CI _T3 vs. T1_, 0.31 to 0.67; p for trend < 0.001) and model III (OR, 0.53; 95% CI _T3 vs. T1_, 0.35 to 0.79; p for trend=0.003). A high intake of antioxidant dietary factors was significantly inversely associated with GC risk in model II (OR, 0.48; 95% CI _T3 vs. T1_, 0.33 to 0.71; p for trend < 0.001) and model III (OR, 0.54; 95% CI _T3 vs. T1_, 0.36 to 0.81; p for trend=0.004). Similar results were obtained for the antioxidant lifestyle factor of regular exercise in model II (OR, 0.53; 95% CI _T3 vs. T1_, 0.39 to 0.72; p for trend < 0.001) and model III (OR, 0.52; 95% CI _T3 vs. T1_, 0.37 to 0.73; p for trend < 0.001).

### Association between the oxidative balance score and gastric cancer risk by histological subtype

The associations between the OBS and GC risk showed different results according to the histological subtype of GC, as shown in Table 4. In patients with the intestinal subtype, the TOBS showed an inverse association with GC risk in both model II (OR, 0.22; 95% CI _T3 vs. T1_, 0.13 to 0.39; p for trend < 0.001) and model III (OR, 0.27; 95% CI _T3 vs. T1_, 0.15 to 0.48; p for trend < 0.001). The highest scores for pro-oxidant lifestyle factors were significantly associated with an increased risk of GC in model II (OR, 2.95; 95% CI _T3 vs. T1_, 1.77 to 4.90; p for trend < 0.001) and model III (OR, 2.91; 95% CI _T3 vs. T1_, 1.72 to 4.92; p for trend < 0.001). However, the highest total antioxidant score and dietary factors were associated with a reduced risk of GC in model II (total antioxidant factors: OR, 0.30; 95% CI _T3 vs. T1_, 0.17 to 0.54; p for trend < 0.001; antioxidant dietary factors: OR, 0.33; 95% CI _T3 vs. T1_, 0.18 to 0.60; p for trend < 0.001) and model III (total antioxidant factors: OR, 0.34; 95% CI _T3 vs. T1_, 0.19 to 0.62; p for trend < 0.001; antioxidant dietary factors: OR, 0.36; 95% CI _T3 vs. T1_, 0.20 to 0.66; p for trend < 0.001). In the diffuse subtype of GC, antioxidant lifestyle factors showed a significant inverse association with GC risk in model II (OR, 0.51; 95% CI _T3 vs. T1_, 0.33 to 0.77; p for trend= 0.008) and model III (OR, 0.49; 95% CI _T3 vs. T1_, 0.32 to 0.76; p for trend= 0.006). In patients with the mixed subtype, the GC risk was reduced for those with the highest total antioxidant factors and antioxidant dietary factor scores in model II (total antioxidant factors: OR, 0.40; 95% CI _T3 vs. T1_, 0.17 to 0.94; p for trend= 0.033; antioxidant dietary factors: OR, 0.41; 95% CI _T3 vs. T1_, 0.17 to 0.98; p for trend= 0.041).

### Association between oxidative balance score and gastric cancer risk among subjects with *Helicobacter pylori* infection

Table 5 presents the association between the OBS and GC risk in GC patients with *H. pylori* infection. The highest TOBS was associated with a decreased risk of GC in participants with *H. pylori* infection (OR, 0.48; 95% CI _T3 vs. T1_, 0.32 to 0.73; p for trend <0.001). In particular, the total antioxidant factors were significantly associated with GC risk in subjects with *H. pylori* infection (OR, 0.57; 95% CI _T3 vs. T1_, 0.37 to 0.88; p for trend= 0.014). Dietary antioxidants and the relevant lifestyle factors decreased GC risk among the patients who were infected with *H. pylori* (antioxidant dietary factors: OR, 0.58; 95% CI _T3 vs. T1_, 0.37 to 0.89; p for trend= 0.018; antioxidant lifestyle factors: OR, 0.50; 95% CI _T3 vs. T1_, 0.35 to 0.72; p for trend < 0.001). However, no associations between pro-oxidant factors and GC risk were observed in participants with *H. pylori* infection.

## DISCUSSION

In the present study, the combined environmental exposures that contribute to oxidative imbalance were associated with GC risk. According to the subclass analysis of pro-oxidant and antioxidant factors, having a greater number of antioxidant diet and lifestyle factors was associated with a decreased risk of GC. The extent of the risk was particularly altered by histological subtype and *H. pylori* infection.

The OBS was developed based on the balance of individual pro-oxidant and antioxidant exposures driven by the oxidative stress pathway, as reported by Goodman et al. [13]. Despite the lack of a previous study on the relationship between GC and the oxidative balance fraction, previous studies examining risk factors for GC support our findings. Among lifestyle factors, substantial evidence has strongly suggested that the risk of GC is increased with alcohol consumption, smoking, and being overweight [26]. Consuming foods preserved by salting and N-nitroso compounds derived from processed meat are some of the pro-oxidant factors associated with an increased risk of GC [25]. In addition, little or no fruit intake as a source of various vitamins increased the risk of GC, although limited evidence is available and the conclusions remain unclear [25,26]. However, numerous studies have reported that the consumption of vitamin C derived from fruits is associated with a lower GC risk [25,27]. Our previous studies observed the associations of GC risk with carotenoids and flavonoids obtained from consuming fruits and vegetables as antioxidant components of the OBS [28,29]. In the comprehensive assessment conducted in this study, a predominance of antioxidant diet and lifestyle factors over pro-oxidant factors was found to be associated with a reduced GC risk. In terms of dietary modification, a prospective cohort study indicated that a predominance of antioxidant factors in the oxidative balance plays a role in reducing cancer-related mortality [30].

As relates to the rationale for using individual antioxidant and pro-oxidant factors to construct the OBS, a number of studies have supported the inclusion of these components based on the biological mechanisms of oxidation-reduction reactions [16]. The production and scavenging of ROS derived from oxidative stress are crucial for the pathophysiology of cancer, contributing to cell survival and oncogenic gene expression [31]. Excessive levels of ROS and hydrogen peroxide increase tumor cell proliferation through oncogenic activation and promote differentiation and cell division via stimulation of growth factors, leading to oxidatively induced DNA damage [31,32]. Cancer cell functions are affected by ROS-mediated signaling pathways and the production of antioxidant proteins to regulate the balance between them [33]. To neutralize the extra ROS, a scavenging system that includes enzymatic antioxidants, such as superoxide dismutase, glutathione peroxidase, glutathione reductase, peroxiredoxin, thioredoxin, and catalase, as well as non-enzymatic hydrophilic or lipophilic radical antioxidants, is important to maintain cellular redox homeostasis [34]. A number of studies have suggested that both endogenously synthesized antioxidants (e.g., enzymes) and exogenous dietary antioxidants (e.g., flavonoids, carotenoids, vitamins and minerals) scavenge ROS in tumor cells [34,35]. Despite the beneficial effects of antioxidants, modulation of the balance between pro-oxidants and antioxidants, along with redox homeostasis, are important in carcinogenesis [36]. Regarding the risk of GC, the disruption of normal cellular homeostasis by an imbalance in oxidative stress gives rise to inflammatory responses induced by environmental pollution, radiation, smoking, alcohol consumption, non-steroidal anti-inflammatory drugs, certain foods, and *H. pylori* infection [37,38]. Although the role of ROS in the pathogenesis of GC remains unclear, antioxidant defense systems exert a considerable effect on *H. pylori* gastritis by removing free radicals and inhibiting oxidation to regulate proinflammatory cytokine production, inflammatory responses, and cell death [37]. With respect to *H. pylori* infection, a more antioxidant diet and lifestyle were associated with a decreased risk of GC in *H. pylori* infection.

We observed different effects of the OBS and its subclasses according to the histological subtype of GC categorized according to the Lauren classification. With respect to the etiological risk factors depending on the histological subtype of GC, the intestinal type is often affected by environmental factors, while the diffuse type is more often associated with genetic susceptibility [39- 41]. Several studies have reported that intestinal GC commonly occurs in individuals with *H. pylori* infection along with dietary (e.g., intake of salt, fruits, and vegetables) and lifestyle (e.g., smoking and alcohol consumption) factors through a multistage process of gastric carcinogenesis [42-44]. Our previous study examined the association between the incidence of GC and the dietary antioxidant capacity based on the oxygen radical absorbance capacity [45]. Several studies have indicated that the free radicals ingested through cigarette smoking and alcohol consumption that induces nitric oxide synthesis, dietary iron consumption involved in the Fenton reaction, and N-nitroso compounds consumed in processed meat are pro-oxidant factors resulting in excess production of ROS, and their accumulation in tissues induces oxidative stress, *H. pylori*-induced gastric inflammation, and DNA damage, culminating in gastric carcinogenesis [37,46]. In this study, we observed similar results in that pro-oxidant lifestyle factors increased the risk for intestinal GC. Additionally, a predominance of antioxidant dietary and lifestyle factors decreased the risk in subjects with intestinal GC. In contrast, the diffuse type of GC results from gastritis linked to chronic inflammation, bypassing the intermediate steps of the carcinogenetic process [47]. Regarding the etiological risk factors for diffuse GC, the present study showed that more antioxidant lifestyle factors were associated with a decreased risk of diffuse GC. These variations suggest that the effect of oxidants, either pro-oxidant or antioxidant factors, may differ slightly from the specific oxidative stress-induced molecular mechanisms underlying the development of different histological subtypes of GC.

The major strength of this study is the recruitment of patients with GC and the assessment of the association between GC risk and the OBS, because no previous studies have estimated GC risk with the OBS. Given the different environmental factors involved in GC etiology depending on the histological subtype, this method facilitates assessments of a relatively comprehensive range of components, ranging from diet to lifestyle factors, that are linked to oxidative stress in the 2 main types of GC. Individual scores for each antioxidant and pro-oxidant factor were measured to identify the combined effects of oxidative stress, unlike other studies [16]. However, our study has some limitations. In this hospital-based case-control study, we recruited controls from a health checkup program who had a healthier lifestyle than the general population. Selection bias might have affected the results. Dietary information obtained using the SQFFQ, which depends on personal memory, may be a source of recall bias. The dietary assessment tool (SQFFQ) was developed using a database of cultural dietary behaviors but not specific nutrients (e.g., carotenoids and flavonoids). Measurement error may have potentially influenced the constructed OBS since the use of dietary supplements for nutrient intake and medication could not be estimated. Although we used the OBS with equal weights, among a variety of OBS calculation methods, to investigate the association with GC risk, the results may plausibly differ from other OBS calculation methods for various types of cancer. An OBS-equal weight method is needed for validation using nutrient databases from other countries in studies with larger sample sizes to improve the validity of our findings. Moreover, the number of subjects who were not infected with *H. pylori* was relatively small for the stratified analysis; in particular, a limited number of patients with GC were not infected with *H. pylori*. A large sample of individuals both negative and positive for *H. pylori* infection is required to examine the associations. Further studies are needed to measure biological markers and validate the results of the present study through a prospective cohort study with a large sample.

In conclusion, significant associations were observed between GC risk and a combined score based on diet and lifestyle factors underlying the oxidative stress mechanism. A predominance of antioxidant factors from the diet and lifestyle decreased GC risk compared to pro-oxidant factors. The effect of antioxidant components may differ by individual *H. pylori* infection and histological subtype, and prospective studies with larger sample sizes are needed to validate our findings. Furthermore, an understanding of the role of the antioxidant potential in gastric carcinogenesis might provide more specific strategies for the prevention of GC in daily life.

## Figures and Tables

**Figure f2-epih-44-e2022089:**
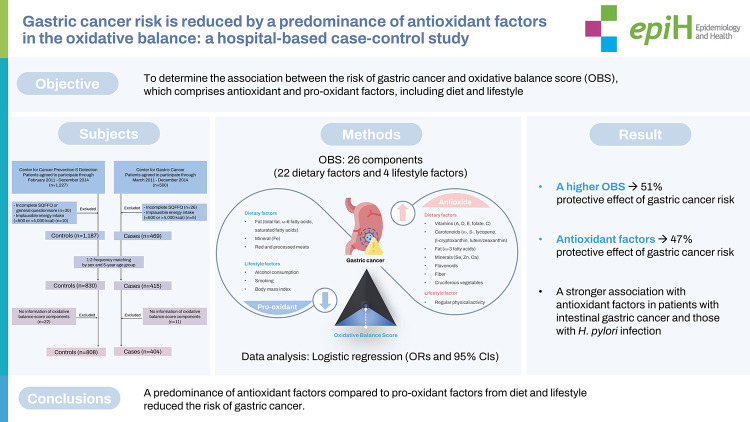


**Table 1.** General characteristics of the study subjects

Characteristics		Controls (n=808)	Cases (n=404)	p-value^1^
Age (yr)		53.75±9.01	53.84±9.00	0.860
Sex				0.970
	Male	535 (66.2)	268 (66.3)	
	Female	273 (33.8)	136 (33.7)	
BMI (kg/m^2^)		24.11±2.81	23.99±2.90	0.500
Education				<0.001
	Middle school or less	117 (14.5)	138 (34.2)	
	High school	243 (30.1)	169 (41.8)	
	College or more	416 (51.5)	96 (23.8)	
Occupation				<0.001
	Professional or administrative	153 (18.9)	69 (17.1)	
	Office, sales or service	256 (31.7)	118 (29.2)	
	Laborer or agricultural	127 (15.7)	103 (25.5)	
	Others or unemployed	269 (33.3)	113 (28.0)	
Household income (10,000 KRW/mo)				<0.001
	<200	144 (17.8)	127 (31.4)	
	200-<400	332 (41.1)	145 (35.9)	
	>400	267 (33.0)	95 (23.5)	
*Helicobacter pylori* infection				<0.001
	Positive	478 (59.2)	371 (91.8)	
	Negative	308 (38.1)	33 (8.2)	
First-degree family history of GC				<0.001
	Yes	100 (12.4)	80 (19.8)	
	No	706 (87.4)	323 (80.0)	
Alcohol consumption				0.200
	Current	526 (65.1)	248 (61.4)	
	Former	59 (7.3)	41 (10.2)	
	None	223 (27.6)	115 (28.5)	
Smoking status				<0.001
	Current	160 (19.8)	126 (31.2)	
	Former	281 (34.8)	119 (29.5)	
	None	367 (45.4)	159 (39.4)	
Regular physical activity				<0.001
	Yes	454 (56.2)	146 (36.1)	
	No	351 (43.4)	258 (63.9)	
METs/wk^2^		2,673.59±2,831.19	2,508.85±3,254.87	0.390
Total energy intake (kcal/day)		1,714.64±542.21	1,929.86±616.61	<0.001
Total oxidative balance score^3^		25.86±8.30	23.76±8.52	<0.001
Total pro-oxidant factors^3^		7.91±3.64	8.17±3.56	0.250
	Pro-oxidant dietary factors^3^	5.00±3.04	5.37±3.00	0.043
	Pro-oxidant lifestyle factors	2.91±1.66	2.80±1.69	0.250
Total antioxidant factors^3^		17.95±9.07	15.60±8.84	<0.001
	Antioxidant dietary factors^3^	16.94±9.00	14.76±8.70	<0.001
	Antioxidant lifestyle factor	1.01±0.81	0.83±0.84	<0.001
Histological subtype of GC (Lauren’s classification)				
	Intestinal	-	156 (38.6)	-
	Diffuse	-	157 (38.9)	-
	Mixed	-	58 (14.4)	-
	Intermediate	-	3 (0.7)	-
	Missing		30 (7.4)	-

Values are presented as mean±standard deviation or number (%). BMI, body mass index; GC, gastric cancer; KRW, Korean won; MET, metabolic equivalent for task.

1 Using the chi-square test for categorical variables and the t-test for continuous variables.

2 METs were assessed using the Mann-Whitney U test.

3 All dietary components (food and nutrients) were adjusted for total energy intake.

**Table 2.** Comparison of individual components of the oxidative balance score

Variables				Controls (n=808)	Cases (n=404)	p-value^1^
Total pro-oxidant factors						
	Pro-oxidant dietary factors	Fat (g/day)^2^	Total fat	20.53±7.84	19.94±8.19	0.220
			PUFAs (ω-6 fatty acids)	4.38±1.44	4.28±1.37	0.240
			Saturated fatty acid	8.64±4.14	8.30±3.74	0.140
		Mineral (mg/day)^2^	Iron	14.00±3.79	13.32±3.72	0.003
		Food (g/day)^2^	Red and processed meats	58.28±38.48	56.66±36.32	0.480
	Pro-oxidant lifestyle factors	Alcohol consumption	Current	526 (65.1)	248 (61.4)	0.190
			Ex-drinker	59 (7.3)	41 (10.2)	
			None	223 (27.6)	115 (28.5)	
		Smoking status	Current	160 (19.8)	126 (31.2)	<0.001
			Ex-smoker	281 (34.8)	119 (29.5)	
			None	367 (45.4)	159 (39.4)	
		BMI (kg/m^2^)	Obese (≥25.0)	266 (32.9)	132 (32.7)	0.950
			Overweight (23.0-24.9)	249 (30.8)	122 (30.2)	
			Normal (18.5-22.9)	293 (36.3)	150 (37.1)	
Total antioxidant factors						
	Antioxidant dietary factors	Vitamins^2^	Vitamin A (μg RE/day)	625.17±331.85	580.62±324.20	0.027
			Vitamin D (μg/day)	2.78±1.95	2.38±1.56	<0.001
			Vitamin E (mg/day)	9.07±2.55	8.53±2.35	<0.001
			Folate (μg/day)	509.44±169.48	484.73±173.49	0.018
			Vitamin C (mg/day)	107.78±55.87	96.04±50.33	<0.001
		Carotenoids (μg/day)^2^	α-carotene	941.53±910.44	924.67±972.38	0.770
			β-carotene	5,068.29±3,297.74	4,631.04±3,015.62	0.021
			Lycopene	2,198.45±3,851.47	1,444.02±2,152.03	<0.001
			β-cryptoxanthin	390.77±429.91	328.77±359.94	0.008
			Lutein/zeaxanthin	3,536.59±2,612.98	3,449.36±2,772.09	0.590
		Fat (g/dy)^2^	PUFAs (ω-3 fatty acids)	0.61±0.39	0.57±0.30	0.031
		Minerals^2^	Selenium (μg/day)	97.04±14.69	97.60±15.52	0.540
			Zinc (mg/day)	10.21±1.61	10.19±2.96	0.900
			Calcium (mg/day)	475.28±194.38	441.60±178.94	0.004
		Flavonoids (mg/day)^2^		106.22±59.59	94.26±59.19	0.001
		Fiber (g/day)^2^		20.77±6.34	19.70±6.51	0.006
		Food (g/day)^2^	Cruciferous vegetables	36.21±29.49	31.78±26.81	0.009
	Antioxidant lifestyle factors	Regular physical activity (METs/wk)^3^	<990	258 (31.9)	183 (45.3)	<0.001
			990-2,970	283 (35.0)	106 (26.2)	
			≥2,970	267 (33.0)	115 (28.5)	

Values are presented as mean±standard deviation or number (%). BMI, body mass index; METs, metabolic equivalents for task; PUFAs, polyunsaturated fatty acids.

1 Using the t-test between the cases and controls.

2 All dietary components (food and nutrients) were adjusted for total energy intake.

3 METs were assessed using the Mann-Whitney U test.

**Table 3.** Association between the oxidative balance score and gastric cancer risk^1^

Variables		No. of controls/cases	Model I	Model II	Model III
TOBS^2^	T1	242/165	1.00 (reference)	1.00 (reference)	1.00 (reference)
	T2	268/125	0.68 (0.51, 0.91)	0.60 (0.44, 0.83)	0.65 (0.46, 0.92)
	T3	298/114	0.56 (0.42, 0.75)	0.41 (0.28, 0.58)	0.49 (0.33, 0.71)
	p for trend		<0.001	<0.001	<0.001
Total pro-oxidant factors^2^	T1	292/151	1.00 (reference)	1.00 (reference)	1.00 (reference)
	T2	280/153	1.06 (0.80, 1.40)	1.29 (0.95, 1.75)	1.31 (0.95, 1.81)
	T3	236/100	0.82 (0.60, 1.11)	0.99 (0.71, 1.37)	0.93 (0.65, 1.32)
	p for trend		0.280	0.830	0.920
Pro-oxidant dietary factors^2^	T1	284/157	1.00 (reference)	1.00 (reference)	1.00 (reference)
	T2	317/155	0.88 (0.67, 1.16)	1.02 (0.76, 1.37)	1.02 (0.75, 1.40)
	T3	207/92	0.80 (0.59, 1.10)	0.89 (0.63, 1.25)	0.85 (0.59, 1.22)
	p for trend		0.160	0.570	0.460
Pro-oxidant lifestyle factors	T1	298/135	1.00 (reference)	1.00 (reference)	1.00 (reference)
	T2	335/173	1.14 (0.87, 1.50)	1.21 (0.90, 1.62)	1.26 (0.92, 1.73)
	T3	175/96	1.21 (0.88, 1.67)	1.49 (1.05, 2.11)	1.41 (0.98, 2.02)
	p for trend		0.210	0.028	0.050
Total antioxidant factors^2^	T1	263/162	1.00 (reference)	1.00 (reference)	1.00 (reference)
	T2	265/141	0.86 (0.65, 1.15)	0.61 (0.61, 1.15)	0.90 (0.64, 1.26)
	T3	280/101	0.59 (0.43, 0.79)	0.46 (0.31, 0.67)	0.53 (0.35, 0.79)
	p for trend		<0.001	<0.001	0.003
Antioxidant dietary factors^2^	T1	258/158	1.00 (reference)	1.00 (reference)	1.00 (reference)
	T2	278/146	0.86 (0.65, 1.14)	0.82 (0.60, 1.12)	0.90 (0.64, 1.26)
	T3	272/100	0.60 (0.44, 0.81)	0.48 (0.33, 0.71)	0.54 (0.36, 0.81)
	p for trend		0.001	<0.001	0.004
Antioxidant lifestyle factors	T1	258/183	1.00 (reference)	1.00 (reference)	1.00 (reference)
	T2	283/106	0.53 (0.39, 0.71)	0.52 (0.38, 0.71)	0.54 (0.39, 0.75)
	T3	267/115	0.61 (0.46, 0.81)	0.53 (0.39, 0.72)	0.52 (0.37, 0.73)
	p for trend		0.005	<0.001	<0.001

Values are presented as odds ratio (95% confidence interval). TOBS, total oxidative balance score; T, tertile.

1 Model I: crude; Model II: adjusted for education, income, sodium intake, and first-degree of family history of gastric cancer; Model III: additionally adjusted for *Helicobacter pylori* infection.

2 All dietary components (food and nutrients) were adjusted for total energy intake.

**Table 4.** Association between the oxidative balance score and gastric cancer risk by histological subtype^1^

Variables			No. of controls/cases	Model I	Model II	Model III
Intestinal						
	TOBS^2^	T1	242/76	1.00 (reference)	1.00 (reference)	1.00 (reference)
		T2	268/48	0.57 (0.38, 0.85)	0.46 (0.29, 0.73)	0.49 (0.30, 0.78)
		T3	298/32	0.34 (0.22, 0.53)	0.22 (0.13, 0.39)	0.27 (0.15, 0.48)
		p for trend		<0.001	<0.001	<0.001
	Total pro-oxidant factors^2^	T1	292/54	1.00 (reference)	1.00 (reference)	1.00 (reference)
		T2	280/57	1.10 (0.73, 1.65)	1.33 (0.85, 2.08)	1.31 (0.83, 2.09)
		T3	236/45	1.03 (0.67, 1.59)	1.34 (0.83, 2.15)	1.26 (0.77, 2.05)
		p for trend		0.840	0.200	0.320
	Pro-oxidant dietary factors^2^	T1	284/61	1.00 (reference)	1.00 (reference)	1.00 (reference)
		T2	317/57	0.84 (0.56, 1.24)	0.96 (0.63, 1.48)	0.96 (0.62, 1.50)
		T3	207/38	0.86 (0.55, 1.33)	1.00 (0.61, 1.63)	0.94 (0.57, 1.56)
		p for trend		0.420	0.970	0.810
	Pro-oxidant lifestyle factors	T1	298/41	1.00 (reference)	1.00 (reference)	1.00 (reference)
		T2	335/67	1.45 (0.96, 2.21)	1.76 (1.11, 2.79)	1.81 (1.12, 2.92)
		T3	175/48	1.99 (1.26, 3.15)	2.95 (1.77, 4.90)	2.91 (1.72, 4.92)
		p for trend		0.004	<0.001	<0.001
	Total antioxidant factors^2^	T1	263/73	1.00 (reference)	1.00 (reference)	1.00 (reference)
		T2	265/50	0.68 (0.46, 1.01)	0.62 (0.39, 0.97)	0.65 (0.40, 1.04)
		T3	280/33	0.43 (0.27, 0.66)	0.30 (0.17, 0.54)	0.34 (0.19, 0.62)
		p for trend		<0.001	<0.001	<0.001
	Antioxidant dietary factors^2^	T1	258/71	1.00 (reference)	1.00 (reference)	1.00 (reference)
		T2	278/52	0.68 (0.46, 1.01)	0.63 (0.40, 0.99)	0.68 (0.42, 1.08)
		T3	272/33	0.44 (0.28, 0.69)	0.33 (0.18, 0.60)	0.36 (0.20, 0.66)
		p for trend		<0.001	<0.001	<0.001
	Antioxidant lifestyle factors	T1	258/69	1.00 (reference)	1.00 (reference)	1.00 (reference)
		T2	283/38	0.50 (0.33, 0.77)	0.48 (0.30, 0.77)	0.50 (0.31, 0.82)
		T3	267/49	0.69 (0.46, 1.03)	0.59 (0.38, 0.92)	0.58 (0.36, 0.91)
		p for trend		0.190	0.060	0.050
Diffuse						
	TOBS^2^	T1	242/55	1.00 (reference)	1.00 (reference)	1.00 (reference)
		T2	268/51	0.84 (0.55, 1.27)	0.78 (0.50, 1.23)	0.83 (0.52, 1.32)
		T3	298/51	0.75 (0.50, 1.14)	0.64 (0.39, 1.05)	0.77 (0.46, 1.28)
		p for trend		0.190	0.080	0.320
	Total pro-oxidant factors^2^	T1	292/61	1.00 (reference)	1.00 (reference)	1.00 (reference)
		T2	280/63	1.08 (0.73, 1.59)	1.34 (0.89, 2.03)	1.34 (0.87, 2.05)
		T3	236/33	0.67 (0.42,1.06)	0.79 (0.49, 1.28)	0.73 (0.45, 1.20)
		p for trend		0.150	0.580	0.410
	Pro-oxidant dietary factors^2^	T1	284/61	1.00 (reference)	1.00 (reference)	1.00 (reference)
		T2	317/63	0.93 (0.63,1.36)	1.06 (0.70, 1.60)	1.08 (0.70, 1.65)
		T3	207/33	0.74 (0.47, 1.18)	0.81 (0.50, 1.32)	0.76 (0.46, 1.26)
		p for trend		0.230	0.500	0.400
	Pro-oxidant lifestyle factors	T1	298/61	1.00 (reference)	1.00 (reference)	1.00 (reference)
		T2	335/67	0.98 (0.67, 1.43)	1.04 (0.70, 1.53)	1.07 (0.71, 1.61)
		T3	175/29	0.81 (0.50, 1.31)	0.97 (0.59, 1.60)	0.92 (0.55, 1.53)
		p for trend		0.490	0.990	0.910
	Total antioxidant factors^2^	T1	263/58	1.00 (reference)	1.00 (reference)	1.00 (reference)
		T2	265/56	0.96 (0.64, 1.44)	1.00 (0.64, 1.54)	1.08 (0.69, 1.71)
		T3	280/43	0.70 (0.45, 1.07)	0.65 (0.38, 1.09)	0.77 (0.45, 1.32)
		p for trend		0.100	0.110	0.370
	Antioxidant dietary factors^2^	T1	258/55	1.00 (reference)	1.00 (reference)	1.00 (reference)
		T2	278/60	1.01 (0.68, 1.52)	1.04 (0.67, 1.60)	1.15 (0.73, 1.80)
		T3	272/42	0.72 (0.47, 1.12)	0.70 (0.41, 1.18)	0.80 (0.46, 1.38)
		p for trend		0.160	0.200	0.470
	Antioxidant lifestyle factors	T1	258/78	1.00 (reference)	1.00 (reference)	1.00 (reference)
		T2	283/35	0.41 (0.27, 0.63)	0.41 (0.26, 0.64)	0.42 (0.27, 0.67)
		T3	267/44	0.55 (0.36, 0.82)	0.51 (0.33, 0.77)	0.49 (0.32, 0.76)
		p for trend		0.016	0.008	0.006
Mixed						
	TOBS^2^	T1	242/17	1.00 (reference)	1.00 (reference)	1.00 (reference)
		T2	268/18	0.96 (0.48, 1.90)	0.72 (0.35, 1.51)	0.78 (0.37, 1.65)
		T3	298/23	1.10 (0.57, 2.10)	0.52 (0.23, 1.16)	0.64 (0.28, 1.46)
		p for trend		0.740	0.110	0.290
	Total pro-oxidant factors^2^	T1	292/26	1.00 (reference)	1.00 (reference)	1.00 (reference)
		T2	280/20	0.80 (0.44, 1.47)	0.90 (0.48, 1.71)	1.00 (0.52, 1.93)
		T3	236/12	0.57 (0.28, 1.16)	0.57 (0.27, 1.18)	0.56 (0.26, 1.18)
		p for trend		0.120	0.150	0.160
	Pro-oxidant dietary factors^2^	T1	284/27	1.00 (reference)	1.00 (reference)	1.00 (reference)
		T2	317/20	0.66 (0.36, 1.21)	0.64 (0.34, 1.22)	0.70 (0.36, 1.34)
		T3	207/11	0.56 (0.27, 1.15)	0.48 (0.23, 1.04)	0.47 (0.22, 1.03)
		p for trend		0.080	0.050	0.050
	Pro-oxidant lifestyle factors	T1	298/23	1.00 (reference)	1.00 (reference)	1.00 (reference)
		T2	335/24	0.93 (0.51, 1.68)	1.01 (0.54, 1.87)	1.04 (0.55, 1.98)
		T3	175/11	0.81 (0.39, 1.71)	1.08 (0.50, 2.35)	1.02 (0.46, 2.27)
		p for trend		0.610	0.880	0.920
	Total antioxidant factors^2^	T1	263/18	1.00 (reference)	1.00 (reference)	1.00 (reference)
		T2	265/23	1.27 (0.67, 2.41)	0.91 (0.45, 1.83)	1.01 (0.49, 2.08)
		T3	280/17	0.89 (0.45, 1.76)	0.40 (0.17, 0.94)	0.49 (0.20, 1.17)
		p for trend		0.730	0.033	0.100
	Antioxidant dietary factors^2^	T1	258/18	1.00 (reference)	1.00 (reference)	1.00 (reference)
		T2	278/23	1.19 (0.63, 2.25)	0.85 (0.42, 1.71)	0.98 (0.47, 2.02)
		T3	272/17	0.90 (0.45, 1.78)	0.41 (0.17, 0.98)	0.48 (0.20, 1.17)
		p for trend		0.750	0.041	0.100
	Antioxidant lifestyle factors	T1	258/21	1.00 (reference)	1.00 (reference)	1.00 (reference)
		T2	283/22	0.96 (0.51, 1.78)	1.04 (0.54, 1.99)	1.10 (0.56, 2.15)
		T3	267/15	0.69 (0.35, 1.37)	0.62 (0.30, 1.27)	0.60 (0.29, 1.25)
		p for trend		0.260	0.150	0.130

Values are presented as odds ratio (95% confidence interval). TOBS, total oxidative balance score; T, tertile.

1 Model I: crude; Model II: adjusted for education, income, sodium intake, and first-degree of family history of gastric cancer; Model III: additionally adjusted for *Helicobacter pylori* infection.

2 All dietary components (food and nutrients) were adjusted for total energy intake.

**Table 5.** Association between the oxidative balance score and gastric cancer risk among subjects with *Helicobacter pylori* infection^1^

Variables		No. of controls/cases	Model I	Model II
TOBS^2^	T1	160/155	1.00 (reference)	1.00 (reference)
	T2	162/112	0.71 (0.52, 0.99)	0.60 (0.42, 0.87)
	T3	156/104	0.69 (0.49, 0.96)	0.48 (0.32, 0.73)
	p for trend		0.028	<0.001
Total pro-oxidant factors^2^	T1	172/137	1.00 (reference)	1.00 (reference)
	T2	161/139	1.08 (0.79, 1.49)	1.32 (0.93, 1.87)
	T3	145/95	0.82 (0.58, 1.16)	0.97 (0.67, 1.41)
	p for trend		0.350	0.910
Pro-oxidant dietary factors^2^	T1	171/141	1.00 (reference)	1.00 (reference)
	T2	184/144	0.95 (0.70,1.30)	1.10 (0.78, 1.54)
	T3	123/86	0.85 (0.60,1.21)	0.88 (0.60, 1.30)
	p for trend		0.380	0.670
Pro-oxidant lifestyle factors	T1	173/124	1.00 (reference)	1.00 (reference)
	T2	189/158	1.17 (0.85, 1.60)	1.23 (0.88, 1.72)
	T3	116/89	1.07 (0.75, 1.53)	1.37 (0.93, 2.02)
	p for trend		0.530	0.100
Total antioxidant factors^2^	T1	174/148	1.00 (reference)	1.00 (reference)
	T2	154/130	0.99 (0.72, 1.37)	0.94 (0.65, 1.35)
	T3	150/93	0.73 (0.52, 1.02)	0.57 (0.37, 0.88)
	p for trend		0.080	0.014
Antioxidant dietary factors^2^	T1	171/144	1.00 (reference)	1.00 (reference)
	T2	157/135	1.02 (0.74, 1.41)	0.96 (0.67, 1.38)
	T3	150/92	0.73 (0.52, 1.03)	0.58 (0.37, 0.89)
	p for trend		0.080	0.018
Antioxidant lifestyle factors	T1	154/169	1.00 (reference)	1.00 (reference)
	T2	164/97	0.54 (0.39, 0.75)	0.54 (0.38, 0.77)
	T3	160/105	0.60 (0.43, 0.83)	0.50 (0.35, 0.72)
	p for trend		0.009	<0.001

Values are presented as odds ratio (95% confidence interval). TOBS, total oxidative balance score; T, tertile.

1 Model I: crude; Model II: adjusted for education, income, sodium intake, and first-degree of family history of gastric cancer.

2 All dietary components (food and nutrients) were adjusted for total energy intake.

## SUPPLEMENTARY MATERIALS

Supplementary Material 1. Oxidative balance score assignment scheme
