# Supplementary material for: Gastric cancer risk is reduced by a predominance of antioxidant factors in the oxidative balance: a hospital-based case-control study in Korea
Source: Epidemiol Health. 2022 Oct 17;44:e2022089. doi: 10.4178/epih.e2022089 (PMC10185973; doi:10.4178/epih.e2022089)
Supplement: Supplementary Material 1. — Oxidative balance score assignment scheme [file epih-44-e2022089-Supplementary-1.docx]

**Supplementary Material 1. Oxidative balance score assignment scheme**

| **Component** | | | **Scoring assignment** | | |
| --- | --- | --- | --- | --- | --- |
|  |  |  | **0** | **1** | **2** |
| **Pro-oxidant factors** |  |  |  |  |  |
| Pro-oxidant  dietary factors | Fat | Total fat (g/day) ^a^ | 3rd tertile (High intake) | 2nd tertile (Medium intake) | 1st tertile (Low intake) |
|  |  | PUFAs (ω-6 fatty acids, g/day) ^a^ | 3rd tertile (High intake) | 2nd tertile (Medium intake) | 1st tertile (Low intake) |
|  |  | Saturated fatty acid (g/day) ^a^ | 3rd tertile (High intake) | 2nd tertile (Medium intake) | 1st tertile (Low intake) |
|  | Mineral | Iron (mg/day) ^a^ | 3rd tertile (High intake) | 2nd tertile (Medium intake) | 1st tertile (Low intake) |
|  | Food | Red and processed meats (g/day) ^a^ | 3rd tertile (High intake) | 2nd tertile (Medium intake) | 1st tertile (Low intake) |
| Pro-oxidant  lifestyle factors | Alcohol consumption status | | Current | Ex-drinker | None |
|  | Smoking status | | Current | Ex-smoker | None |
|  | BMI (kg/m^2^) | | Obesity (≥25) | Overweight (23.0-24.9) | Normal (18.5-22.9) |
| **Antioxidant factors** |  |  |  |  |  |
| Antioxidant  dietary factors | Vitamins | Vitamin A (μg RE/day) ^a^ | 1st tertile (Low intake) | 2nd tertile (Medium intake) | 3rd tertile (High intake) |
|  |  | Vitamin D (μg /day) ^a^ | 1st tertile (Low intake) | 2nd tertile (Medium intake) | 3rd tertile (High intake) |
|  |  | Vitamin E (mg/day) ^a^ | 1st tertile (Low intake) | 2nd tertile (Medium intake) | 3rd tertile (High intake) |
|  |  | Folate (μg /day) ^a^ | 1st tertile (Low intake) | 2nd tertile (Medium intake) | 3rd tertile (High intake) |
|  |  | Vitamin C (mg/day) ^a^ | 1st tertile (Low intake) | 2nd tertile (Medium intake) | 3rd tertile (High intake) |
|  | Carotenoids | α-carotene (μg /day) ^a^ | 1st tertile (Low intake) | 2nd tertile (Medium intake) | 3rd tertile (High intake) |
|  |  | β-carotene (μg /day) ^a^ | 1st tertile (Low intake) | 2nd tertile (Medium intake) | 3rd tertile (High intake) |
|  |  | Lycopene (μg /day) ^a^ | 1st tertile (Low intake) | 2nd tertile (Medium intake) | 3rd tertile (High intake) |
|  |  | β-cryptoxanthin (μg /day) ^a^ | 1st tertile (Low intake) | 2nd tertile (Medium intake) | 3rd tertile (High intake) |
|  |  | Lutein/zeaxanthin (μg /day) ^a^ | 1st tertile (Low intake) | 2nd tertile (Medium intake) | 3rd tertile (High intake) |
|  | Fat | PUFAs (ω-3 fatty acids, g/day) ^a^ | 1st tertile (Low intake) | 2nd tertile (Medium intake) | 3rd tertile (High intake) |
|  | Minerals | Selenium (μg /day) ^a^ | 1st tertile (Low intake) | 2nd tertile (Medium intake) | 3rd tertile (High intake) |
|  |  | Zinc (mg/day) ^a^ | 1st tertile (Low intake) | 2nd tertile (Medium intake) | 3rd tertile (High intake) |
|  |  | Calcium (mg/day) ^a^ | 1st tertile (Low intake) | 2nd tertile (Medium intake) | 3rd tertile (High intake) |
|  | Flavonoids (mg/day) ^a^ | | 1st tertile (Low intake) | 2nd tertile (Medium intake) | 3rd tertile (High intake) |
|  | Fiber (g/day) ^a^ | | 1st tertile (Low intake) | 2nd tertile (Medium intake) | 3rd tertile (High intake) |
|  | Food | Cruciferous vegetables (g/day) ^a^ | 1st tertile (Low intake) | 2nd tertile (Medium intake) | 3rd tertile (High intake) |
| Antioxidant  lifestyle factors | Regular physical activity (METs/week) | | 1st tertile (Low METs/week) | 2nd tertile (Medium METs/week) | 3rd tertile (High METs/week) |

BMI, body mass index; METs, metabolic equivalents for task; PUFAs, polyunsaturated fatty acids. ^a^ All dietary components (food and nutrients) were adjusted for total energy intake and then divided into tertiles.
